# Inactivated Polio Vaccine: Its proposed role in the final stages of polio eradication

**DOI:** 10.11604/pamj.2013.14.102.2580

**Published:** 2013-03-13

**Authors:** Robert Davis, Robin Biellik

**Affiliations:** 1 American Red Cross Delegate, Nairobi, Kenya; 2 Consulting Epidemiologist, Geneva, Switzerland

**Keywords:** Inactivated Polio Vaccine, polio eradication, oral polio vaccine, Circulating Vaccine Derived Poliovirus, immunization, financial sustainability

## Emergence of Circulating Vaccine Derived Poliovirus

In 2000, the island of Hispaniola, home to Haiti and the Dominican Republic, was the first place where the existence of circulating vaccine derived poliovirus (cVDPV) was demonstrated [1]. As a live product, oral polio vaccine (OPV), once excreted, could enter the environment, reassort with other enteroviruses, and produce cVDPV [2]. After the investigation of that outbreak, the first of a dozen in the current century, a review of laboratory samples from elsewhere showed that other cVDPV had circulated in countries, notably Egypt [3], and was misclassified as wild poliovirus (WPV) at the time. It was recognized that the extent of cVDPV was large enough to pose a threat to the global eradication programme. In the current century, cVDPV has been found in countries as diverse as China and Madagascar, with three recent cases seen in Kenya, genetically linked to Somalia. A 2008 review of the first eight documented outbreaks, all from countries with poor OPV vaccination coverage, was not reassuring.

“Insofar as eradication of all poliomyelitis is the Global Polio Eradication Initiative (GPEI) target, this will require total cessation of all poliovirus transmission. To describe the problem of vaccine-derived polio as 114 virologically-confirmed cases, worldwide, over some twenty years, gives a very different impression than a description which suggests a minimum of hundreds of thousands, and more likely several million infections by vaccine-derived viruses, some of which became endemic in large populations. It is also possible that other vaccine-derived virus lineages have circulated for limited time periods, but failed to cause any clinical cases and were thus unrecognized”. [4]

By 2008, the World Health Assembly was forced to concede that continued OPV vaccination was incompatible with polio eradication. OPV, and the accompanying risk of cVDPV, is incompatible with polio eradication. Polio eradication cannot be achieved while the use of OPV continues to cause rare cases of vaccine associated paralytic poliomyelitis (VAPP) [5] and cVDPV. Some mechanism is required to prevent VAPP and cVDPV cases while still enjoying the operational and immunological advantages of OPV. This has led to a reconsideration of the potential solution through the careful application of combined inactivated polio vaccine/oral polio vaccine (IPV/OPV) schedules as an interim step towards global cessation of OPV use.

## Proposed solutions

**Figure 1:**
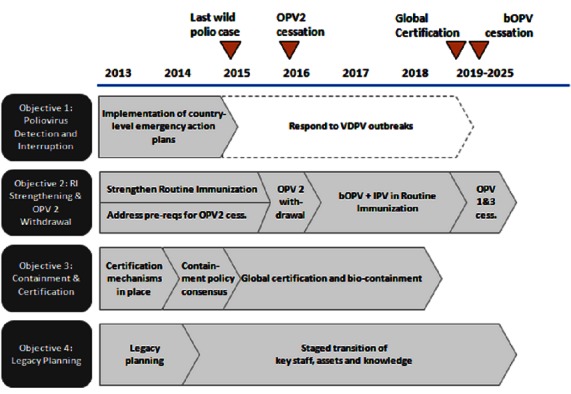
Eradication and endgame strategic plan (source: Polioeradication End Game Strategic Plan)

Current GPEI thinking, set down in the January 2013 document “Polio Eradication and Endgame Strategic Plan (2013-2018)” submitted to the W.H.O. Executive Board [6] (Figure 1) covers the milestones set forth in the figure above. It is proposed that all OPV using countries remove Sabin strain 2 from the environment by switching from trivalent OPV (tOPV) to bivalent OPV (bOPV), which contains only Sabin strains 1 and 3. This also has the advantage of raising vaccine efficacy to types 1 and 3 by removing interference from type 2. It is further proposed to provide all infants and children [7].

### Technical Justifications for Proposed Solutions

The VAPP problem, while of great clinical importance, does not contribute to polio transmission. It is only since 2000, with the emergence of cVPDV, that the need for a move away from OPV, especially from tOPV, has gained urgency. For countries currently using OPV, several solutions to this problem have been proposed:

- Introduction of IPV into the national immunization program, while continuing the use of tOPV or bOPV through the Global Polio Eradication Initiative (South Africa and several Latin American countries have recently done this)
- Cessation of type 2 OPV, which accounts for most cVDPV, while continuing vaccination with types 1 and 3, using (bOPV). The bOPV option, while creating risks of type 2 cVDPV, produces higher seroconversion rates for types 1 and 3, both of which are still endemic as wild type viruses in the world.
- Discontinuation of all OPV, and continuation of IPV, once cessation of WPV and cVDPV is documented [2]

What is known about the risk factors for cVDPV emergence-notably low or declining levels of population immunity against polio-makes the simple cessation of OPV, without complementary measures, an ineffective measure. In the immediate future, while OPV use continues, a switch from tOPV to bOPV will reduce the risk of cVDPV.

### IPV and OPV: characteristics and global implementation to date

From the licensing of the Salk inactivated (killed) polio vaccine (IPV) in 1955 until 1961, there was no debate on the choice of polio vaccine. From 1961 onwards, countries had the choice of OPV and IPV. Led by Sweden, some European countries opted for the inactivated vaccine, which does not produce the vaccine associated paralytic polio (VAPP) associated with OPV. Sweden provided an ideal field for IPV research, since the country never used OPV. After 10 years of IPV administration, a study of vaccinees showed the following:

“More than 95% of subjects under 30 years of age had received 2 or more injections, but the proportion of vaccinated individuals decreased slightly among people over 30 years of age. In the oldest age group questioned (60-70 years) only 20% had been vaccinated. Antibodies to the 3 types of poliovirus were present in more than 95% of the sera in all age groups except two. Samples seronegative to one or more types of virus were found in about 15% of people in the oldest age group and among children vaccinated during the first years of poliovirus vaccination (1957-61).” [8].

The Nordic countries (Scandinavia plus Iceland) all used IPV from the start, in some cases in a mixed IPV/OPV regimen. Writing in 1994, Margareta Böttiger et al. drew the following conclusions from their studies [9].

- Nationwide vaccination with killed vaccine was highly effective
- It is of the utmost importance that the potency of the killed vaccine is high
- Oral vaccine may cause higher rates of vaccine-associated secondary cases than have been reported in general
- When virus is reintroduced into the country, unvaccinated groups are vulnerable. Outbreaks in unvaccinated “pockets” have occurred. This phenomenon, however, has also been experienced in countries using oral vaccine

In Stockholm, both wild poliovirus and vaccine-like polio strains were isolated from the sewage water, indicating a constant import of both types of viruses. Mucosal immunity prevents viral replication in the gut, so that immune individuals excrete little virus after ingesting WPV by mouth. This is critical in the prevention and control of polio transmission leading to outbreaks, by reducing the quantity of WPV excreted into the environment which is available to infect other individuals. A recent review [10] drew the following conclusions about the relative suitability of IPV and OPV for stopping WPV transmission in areas with poor sanitation.

“Individuals vaccinated with OPV were protected against infection and shedding of poliovirus in stool samples collected after challenge compared with unvaccinated individuals…. In contrast, IPV provided no protection against shedding compared with unvaccinated individuals… or when given in addition to OPV, compared with individuals given OPV alone…. There were insufficient studies of nasopharyngeal shedding to draw a conclusion. IPV does not induce sufficient intestinal mucosal immunity to reduce the prevalence of fecal poliovirus shedding after challenge, although there was some evidence that it can reduce the quantity of virus shed.”

Recent evidence, from Cuba and elsewhere, tends to confirm that the development of mucosal immunity following vaccination with IPV does occur, but is limited. Furthermore, the authors of one Cuban study point out that their results “must be seen in the context of Cuba, where there are generally good standards of public health and hygiene. Since WPV was eliminated from the island by 1962, others born after 1962 in Cuba have only vaccine-induced immunity against polio. Whether these results can be generalized to settings with more recent circulation of WPVs remains uncertain” [11]. Most developing countries started polio vaccination after the 1974 launch of the Expanded Programme on Immunization by WHO. Several reasons argued in favor of OPV:

- The oral presentation, making it ideal for administration by lay personnel
- The well documented mucosal immunity from OPV
- The moderate price
- Spreading immunity, with vaccine virus excreted and spread to siblings of vaccinees in places with poor sanitation
- OPVs proven track record in interrupting wild polio transmission in Cuba, then in Brazil and the rest of the western hemisphere

Murdin and colleagues, writing in 1996, reviewed the generally successful experience of industrialized countries with IPV and wrote:

“…the world is divided today into three types of countries. First, in Africa and parts of Asia, there are countries in which wild poliovirus still circulates freely, and in those countries OPV is indicated in order to spread attenuated poliovirus throughout the population and thus to rapidly terminate circulation of wild poliovirus among both vaccinees and non-vaccinees. Second, there are many countries in those same continents and in the Middle East in which wild poliovirus is now uncommon, but where the low immunogenicity of OPV makes it necessary to administer multiple doses. In these countries, additional safety and efficacy can be achieved by substituting DTP-eIPV (enhanced IPV) for DTP to supplement OPV in mixed schedules [12]. Third, countries throughout the Americas and Europe are now free of wild poliovirus, and the only paralytic polio seen is that caused by the vaccine. In these countries, the use of IPV could achieve the same protective effect as OPV, with less morbidity [13].”

Between the WHA resolution of 1988 and 2000, the number of endemic countries with persistent transmission declined from 120 to 25 [14]. Type 2 wild poliovirus was eradicated globally in 1998, leading to the adoption of bivalent and monovalent OPV formulations with higher take rates [15]. By the time of the 1988 WHA resolution, countries using IPV had little polio, so that the polio declines since 88 have happened almost exclusively in countries using OPV.

OPV continued to be favored by most countries as recently as the year 2000. OPV was, for decades, the antigen of choice in countries with suboptimal sanitation and a concomitant high prevalence of enterovirus infections in the gastro-intestinal tracts of infants and children. The skeptical view that OPV could not stop transmission in India [16] was disproven in 2011, when India saw her last case of wild poliovirus. The United States of America started with an IPV based regime in 1955, going over to OPV for general vaccination in 1961. In 1997, the U.S.A. adopted a four dose regime, with two doses of IPV followed by two doses of OPV [17]. In 2000, the U.S.A., coming full circle, returned to the straight IPV schedule of the 1950s. As developing countries were cleared of wild poliovirus, with cVDPV cases coming to the fore, reconsideration was given to the advantages of IPV. Opinions about the feasibility of IPV introduction in different schedules are not unanimous [18]. In particular, South American countries currently free of cVDPV have been reluctant to opt for the more expensive option of IPV. They may regard IPV as an expensive solution to a cVDPV problem which, for them, does not currently exist.

## Introduction of IPV in Sub-Saharan Africa

To date, South Africa is the only country in sub-Saharan Africa to have adopted a combined IPV/OPV schedule. Here are the reasons for its adoption, as set down by one South African author [19].

“South Africa is currently the only country on the African continent using inactivated polio vaccine (IPV) for routine immunization in a sequential schedule in combination with oral polio vaccine (OPV). IPV is a component of an injectable pentavalent vaccine introduced nationwide in April 2009 and administered according to EPI schedule at 6, 10 and 14 weeks with a booster dose at 18 months. OPV is administered at birth and together with the first IPV dose at 6 weeks, which stimulates gut immune system producing a memory IgA response (OPV), followed by IPV to minimize the risk of vaccine associated paralytic polio (VAPP). OPV is also given to all children under 5 years of age as part of regular mass immunizations campaigns. The decision to incorporate IPV into the routine schedule was not based on cost-effectiveness, which it is not. Other factors were taken into account: Firstly, the sequence benefits from the initial mucosal contact with live (vaccine) virus which promotes the IgA response from subsequent IPV, as well as herd immunity from OPV, together with the safety of IPV. Secondly, given the widespread and increasing use of IPV in the developed world, public acceptance of vaccination in general is enhanced in South Africa which is classified as an upper middle income developing country. Thirdly, to address equity concerns because of the growing use of IPV in the private sector. Fourthly, the advent of combination vaccines facilitated the incorporation of IPV into the EPI schedule.”

## Operational and financial sustainability of proposed solutions

National immunization programmes in Africa have, without exception, succeeded in changing vaccination schedules and antigens, especially since 2001, when GAVI began to offer financial support for the introduction of new and underused vaccines. The prerequisites for smooth transitions from old to new schedules are:

- Sufficient funding, internal and external
- Upgrading cold chain capacity at central, provincial, district and field levels, with purchase of additional equipment where indicated
- Retraining of health workers and technicians
- Sensitization of public and professionals

Following a WHA resolution of May 2012, which endorsed the proposed tOPV-bOPV switch and expressed alarm over the then current IPV price of $2.75, the WHO African Region convened an experts’ meeting in Luanda in October 2012 to review options for future use of IPV in the 46 member states of the region. The Luanda meeting considered the following options for IPV introduction:

- Intramuscular (IM) full dose
- Intradermal (ID) fractional dose - under development
- Adjuvanted IM dose - under development

The Luanda meeting expressed a preference for IPV introduction with full dose IM vaccine administered with needle/syringe, with the understanding that adequate funds could be raised. According to the consultation group, a single dose of IPV may be expected to:

- Prevent paralytic polio caused by cVDPV2, Sabin strain 2 or WPV2
- Produce immunological priming with improved response to mOPV2 (monovalent OPV) or IPV, in an outbreak
- Reduce poliovirus transmission through reduced virus titre and duration of fecal virus excretion.
- Accelerate wild poliovirus eradication by boosting of immunity to types 1 and 3 immunity among individuals previously vaccinated with OPV.

The consultation group agreed that the introduction of “at least one dose of IPV in the routine programme” was appropriate and feasible in AFRO. (Unpublished PowerPoint presentation, WHO/AFRO, Luanda, October 2012).

## Proposed Milestones

As the recent (December 2012) murders of vaccinators in Pakistan have shown, there are unpredictable events which may cause the 2013-2018 eradication timeline to slide further. There will be an increase in global expenditure on polio vaccine after the introduction of single dose or two dose IPV regimes in current non-using countries, likely to cost more than $100 million per year in developing countries introducing IPV. For more than three years, the world will be bearing the double burden of OPV and IPV purchases for most developing countries, perhaps with the added burden of IPV campaigns and their associated operational costs if the mere insertion of IPV into the routine schedule does not, without campaigns, achieve sufficient impact.

The January 2013 draft of the global polio eradication endgame plan calls for introduction of at least one dose of IPV into routine immunization by end 2015, with DPT3 targets of 70% and 80% respectively for 2014 and 2015. Achievement of these objectives within the dates proposed means that remaining deficiencies in programme implementation will need to be addressed and effectively resolved.

The introduction of IPV ahead of the bOPV-tOPV switch will be protective if and only if IPV coverage is high. It will be necessary to consider different strategies in developing countries targeted for IPV introduction, distinguishing between those with adequate and inadequate routine service delivery. Moreover, country-specific communications strategies for IPV introduction will be needed. South Africa's seamless transition from OPV to OPV/IPV is the ideal, which might not be reproduced in other countries, especially those which have seen antivaccination movements in the past. In Nigeria, for example, consideration might be given to consecutive IPV introduction in southern and northern states.

### Vaccine Requirements and Availability

A more severe constraint to the implementation of the endgame strategy than financial factors may be the availability of IPV during the period 2015-2018. WHO thinking on IPV production, with several caveats, is reflected in the WHO secretariat's December 2012 report to the WHO Executive Board [20].

“Three manufacturers have agreed to pursue licensure for intradermal delivery of their inactivated poliovirus vaccine for use in emergency situations, and in one case for routine immunization, with a target price US$ 0.50 per dose and a development timeline of 24 - 36 months. Two manufacturers have agreed to develop an inactivated poliovirus vaccine containing an adjuvant, with a target price of between US$ 0.50 and US$ 0.75 per dose and a timeline of 36 - 48 months, contingent in one case upon substantial external support. A third manufacturer is considering the fast-track development of a similar product. Although two manufacturers are planning to develop a low-dose inactivated poliovirus vaccine as part of their respective hexavalent products, neither product will be available during the period of the new strategic plan. WHO continues to support the transfer to developing countries of new production technology for inactivated poliovirus vaccine using Sabin-strain polioviruses. It is expected that such Sabin-strain inactivated poliovirus vaccines will be available during the period of the new strategic plan [21]; however, additional development work is needed to finalize timelines and expected pricing. In parallel to these and other development efforts, and as recommended by the Scientific Advisory Group of Experts on immunization, WHO, UNICEF, the GAVI Alliance and the Bill & Melinda Gates Foundation are establishing a supply and funding strategy for timely introduction of inactivated poliovirus vaccine using existing full-dose products for a transition period if needed.”

IPV is currently produced in four prequalified European facilities, which serve the needs of the <70 countries currently giving IPV. One of these, in the Netherlands, has just been acquired by the Serum Institute of India, which plans to ramp up capacity using existing technology. The current draft endgame plan calls for IPV global availability at <$ per dose by the end of 2015. How quickly large scale production of IPV can be achieved remains to be seen. We will not know with certainty whether the combined production of all IPV producers can meet the needs of 192 countries in time for early IPV introduction for some time yet. In its January 2013 draft endgame plan, GPEI states:

“Recognizing that the development of these new, low-cost IPV options may not meet the optimal timeline for a tOPV-bOPV switch, the GPEI is working with manufacturers, GAVI and stakeholders to develop by mid-2013 a strategy that would allow initial introduction in low and low-middle income countries using existing IPV products at substantially reduced prices, with a subsequent transition to more sustainable, low-cost products as they became available. By 2017 there should be feasible options for safely producing IPV in developing country settings to ensure that all countries have the opportunity to produce IPV for their routine childhood immunization.”

### Financial Commitments

The current (January 2013) GPEI document calls for IPV availability at [22]. That case is almost unanswerable. The partners which have already invested more than US$7 billion in GPEI will want to protect that investment. The costs are great, but the benefits are greater [23].

## Conclusion

Countries, especially endemic and recently endemic countries, will need to step up to the commitment their ministers made 25 years ago to eradicate polio and invest national funds adequately to ensure that this highly desirable public health goal is achieved as soon as possible. The substantial economic and social benefits to developing countries of achieving polio eradication were established many years ago. Donors should sustain their commitments to complement those of developing countries in the long term, to facilitate more efficient planning and implementation of the strategies described in the draft GPEI strategic plan. However, two other large public health programs will compete for partner attention in the current decade: the Measles and Rubella Initiative, if its seeks to target two diseases for eradication, and the Malaria Vaccine Initiative, which may have a licensed and WHO pre-qualified product ready for introduction in developing countries by 2015.
